# Researcher and community partner perspectives on community-engaged research during the COVID-19 pandemic

**DOI:** 10.1017/cts.2025.10090

**Published:** 2025-07-07

**Authors:** Simone C. Frank, Mary E. Grewe, Milenka Jean-Baptiste, Alicia Bilheimer, Alexandra F. Lightfoot, Laura Villa-Torres

**Affiliations:** 1 North Carolina Translational and Clinical Sciences Institute, University of North Carolina at Chapel Hill, Chapel Hill, NC, USA; 2 Gillings School of Global Public Health, University of North Carolina at Chapel Hill, Chapel Hill, NC, USA

**Keywords:** Community-engaged research (CEnR), covid-19 pandemic, virtual engagement, institutional barriers, translational science

## Abstract

**Introduction:**

We sought to explore how the COVID-19 pandemic impacted community-engaged research (CEnR) from both researcher and community partner perspectives, identify challenges and facilitators affecting their experiences, and describe desired supports for CEnR during future health crises.

**Methods:**

We conducted semi-structured, virtual interviews with ten researchers and eight partners who conducted or collaborated on CEnR during the COVID-19 pandemic. Interviews were recorded and transcribed for analysis. We analyzed the transcribed data thematically through an iterative process involving memoing, consensus coding, and reviewing memos and code reports to identify and describe key categories and themes.

**Results:**

Challenges identified were related to wellbeing and personal circumstances, such as feeling burnt out, managing increased caregiving responsibilities, or concern about risk of illness; institutional barriers, such as inflexible and burdensome financial, regulatory, and administrative policies; and virtual engagement, such as distractions, limited Internet access, or difficulty forming relationships online. Facilitators fell into two categories. Foundational factors such as strong existing partnerships, funding, and project-specific circumstances were critical to facilitating CEnR activities. Strategy-based facilitators focused on overcoming challenges and included communication, flexibility, risk mitigation, and utilizing techniques to enhance virtual engagement. Desired supports included flexible funding, resources for navigating research during crises, and increased virtual engagement accessibility and guidance.

**Conclusions:**

By better understanding challenges and facilitators affecting experiences of researchers and community partners during the COVID-19 pandemic, we can develop strategies and resources to better support CEnR partnerships during future crises.

## Introduction

Community-engaged research (CEnR) serves as a vehicle to involve community throughout the research process to ensure that research questions, approaches, findings, and outcomes are informed by and relevant to communities [1]. CEnR is guided by principles that emphasize collaboration, co-learning, mutual benefit, and capacity building for all partners [1]. Additionally, CEnR methods exist along a continuum of engagement [1–3]. At each level of the continuum, researchers and partners may assume different roles and responsibilities on a project, from researchers seeking one-time feedback or input from partners; to researchers and partners developing and conducting a project together; to full partner ownership of a project, in which researchers may play a supporting role to further a community-driven agenda [1–3]. Depending on level of engagement, partners may undertake a variety of activities on a project, such as participating in time-limited engagement studios; serving on advisory boards; conducting study activities as community co-researchers; or serving as Co-Investigators [4]. Many types of research, including translational research, can benefit from CEnR’s flexible approaches to increase the relevance of research for communities and bridge the gap between research and actionable practice and policy [5,6].

CEnR is also well positioned to consider historical, structural, and cultural factors influencing community health, and thus has long held promise as a research approach to address health disparities [5,7]. For instance, the COVID-19 pandemic exacerbated health inequities in the USA, sparking an increased application of CEnR approaches to research initiatives focused on addressing pandemic-related issues [8–13]. Additionally, just as COVID-19 spurred transformation in the research enterprise as a whole – changing the way research is planned, conducted, and disseminated – CEnR approaches were changed by necessity [8,14–16]. Thus, researchers already engaging in CEnR activities had to adapt [17,18]. Given CEnR’s critical role in integrating community voice and perspective in research, which is especially necessary during times of crisis [19], it is important to understand how CEnR partnerships adapted their approaches during COVID-19, the challenges and facilitators they encountered, and how these challenges and facilitators were experienced by researchers and partners at different levels of the CEnR continuum.

Our team within the Patient and Community Engagement in Research (PaCER) Program at the University of North Carolina at Chapel Hill’s Translational and Clinical Sciences Institute sought to better understand researchers’ experiences engaging community partners in health-related research during the peak of the COVID-19 pandemic, as well as community partners’ experiences engaging in research during this time. We aimed to identify challenges and facilitators affecting their experiences, strategies they adopted, adapted, and generated to continue their engagement, and desired supports for conducting CEnR virtually or during future health crises.

## Methods

We utilized a qualitative description methodology and thematic analysis approach to conduct this qualitative research study.

### Recruitment

**Figure 1.**
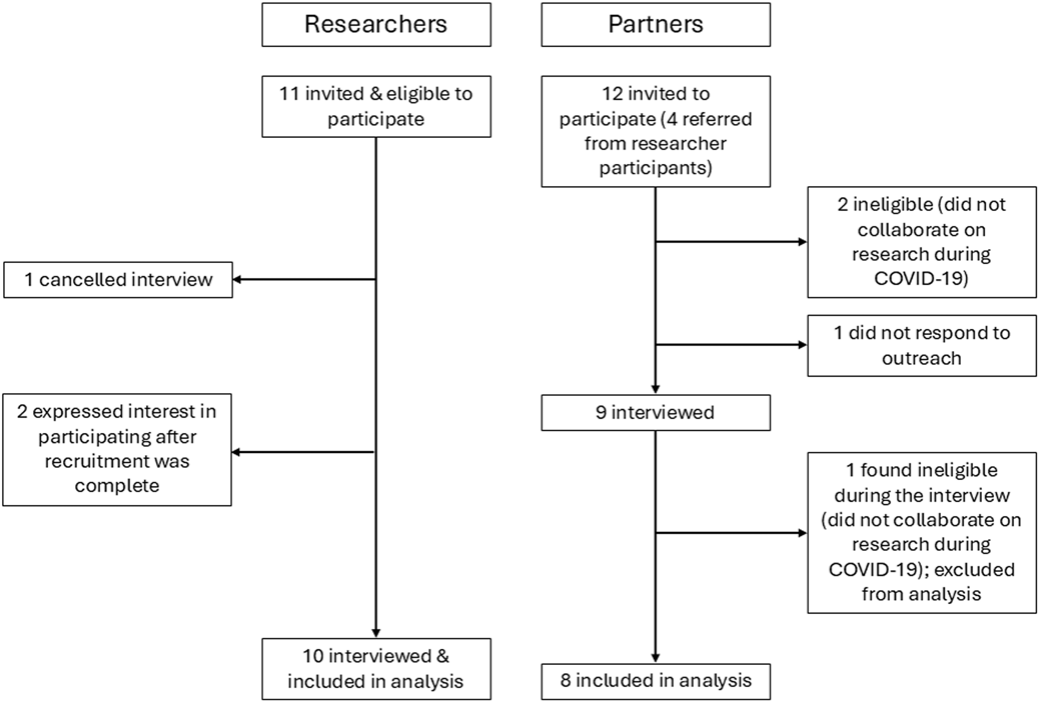
Recruitment flow diagram. Eleven researchers were invited and eligible to participate, one of whom canceled their interview and was not able to reschedule; two additional researchers expressed interest in participating after researcher recruitment was complete. Twelve partners were invited to participate (four were referred from researcher participants); two were ineligible due to not collaborating on research during the COVID-19 pandemic; one did not respond to outreach; and one was identified as ineligible during the interview (did not collaborate on research during the COVID-19 pandemic) and thus was excluded from analysis.

We recruited researchers and community partners (partners) to participate in semi-structured interviews. We utilized purposive and snowball sampling approaches, identifying potential participants based on our knowledge of the field, program and administrative data, and referrals from other researchers and partners. Eligibility criteria included being age 18 or older, identifying as a research investigator, research staff, or partner (e.g., people who work at community organizations, patient advisors, healthcare workers), and having experience collaborating on research conducted through the University of North Carolina at Chapel Hill (UNC-Chapel Hill) during the COVID-19 pandemic (March 2020 and onward). Potential participants were invited to participate via email, and if they agreed, were scheduled for an interview (Figure 1). The study was deemed exempt from full review by the University’s Institutional Review Board.

### Data collection

We conducted interviews between February 2022 and May 2022 with ten researchers and nine partners. One of the nine partners was identified as ineligible during the interview (having not collaborated on research during the COVID-19 pandemic), so was excluded from analysis. All participants provided verbal consent to participate. Interviews took place via Zoom, lasted approximately 30 to 60 minutes, and with participant permission, were audio-recorded for transcription purposes. Interviews were conducted by female Masters or PhD trained members of our team with experience and training in qualitative and CEnR (SF, MG, MJB, LVT). During the semi-structured interviews, we asked participants about their experiences with CEnR projects during the COVID-19 pandemic, focusing on challenges they faced, strategies or other factors that facilitated their engagement, and suggested or desired resources to support CEnR projects in the future (see interview guide in Supplementary Material 1). Following the interview, we verbally delivered a brief demographic survey. Partners were offered a $50 gift card for participating.

### Analysis

Interviews were transcribed by a professional transcription company and reviewed for quality by our team. Each transcript was then reviewed by a team member (SF, MG, LVT), who wrote a short memo summarizing the interview. Memos were used to note initial impressions of and emerging ideas from the data; reflect on the data in relation to the study’s primary aims of identifying challenges, facilitators, strategies, and desired support related to conducting CEnR during COVID-19; and inform the analytic process, specifically codebook development [20,21]. We reviewed and met to discuss the memos after most were written, and created a preliminary codebook consisting of pre-determined codes informed by the study’s aims and inductive codes that emerged during memoing. Then, we began coding the data using Atlas.ti version 9 [22] while completing the remaining memos. Two team members (SF, MG) independently coded the initial transcript, met to discuss their coding until consensus was reached, and updated the codebook, adding emerging codes. This process was repeated for eight transcripts (four researchers and four partners), at which point it was determined that the codebook was stable. One team member (MG) then coded the remaining transcripts, which the other team member (SF) reviewed. One team member (MG) also reviewed initial transcripts coded before the codebook was finalized to ensure that newly developed codes were applied across all transcripts (see final codes and definitions in Supplementary Material 2). During the coding process, we confirmed that we reached data saturation for our research aim.

After coding was finalized, two team members (SF, MG) reviewed summary memos and code reports – focusing on excerpts coded as challenges, facilitators, and desired resources, while noting co-occurring codes – to identify larger patterns and themes in the data. For this paper’s purposes, researcher and partner interviews were analyzed together to understand the overarching participant experience. We summarized these excerpts thematically, a process informed by insights gleaned from memoing. Finally, we reviewed demographic data, collapsing free responses into categories (e.g., “years worked” was asked as an open-ended question, and we collapsed this into categories).

### Quality considerations

We utilized the following strategies to promote credibility, transferability, dependability, and confirmability of our findings: prolonged engagement (in-depth interviews and sufficient time engaging with data), triangulation (interviewees representing different roles, multiple investigators involved), thick description (detailed results, including illustrative quotes), thorough explanation of sampling methods, discussion of results in the context of the literature, sample size sufficient to reach data saturation, iterative data analysis, and use of an audit trail to document the analysis process [23,24].

## Results

### Participants

Ten researchers and eight partners participated in interviews and were included in this analysis. Participants described collaborating on projects varying in scope, methodology, topic, and target population, with many participants discussing multiple projects. Aggregated across all interviews, participants discussed projects that were conducted at local (i.e., within a specific limited geographic community), regional, and national levels; engaged community, patient, provider, and organizational partners; and focused on a variety of chronic health diseases and health needs of various underserved populations. Participants described different levels of engagement, discussing approaches such as advisory boards, community research networks, and community-based participatory research (CBPR) approaches. Participant demographics are displayed in Table 1.

**Table 1.** Participant demographics (N = 18)

Demographic Category	Response Category	Researchers: *n* (%) *N* = 10	Partners: *n* (%) *N* = 8
Gender	Female	8 (80)	7 (87.5)
	Male	2 (20)	1 (12.5)
Race	White	7 (70)	3 (37.5)
	Black / African American	2 (20)	4 (50)
	Asian American	0 (0)	1 (12.5)
	More than one race	1 (10)	0 (0)
Hispanic or Latino/a	No	10 (100)	8 (100)
Years in current position / at university (asked to researchers only)	0 to 5	3 (30)	N/A
	6 to 10	1 (10)	
	11 to 15	2 (20)	
	16 to 20	3 (30)	
	Over 20	1 (10)	
Years worked in community-engaged research projects	0 to 5	1 (10)	2 (25)
	6 to 10	4 (40)	1 (12.5)
	11 to 15	0 (0)	0 (0)
	16 to 20	2 (20)	1 (12.5)
	Over 20	3 (30)	4 (50)

### Challenges

Participants described CEnR challenges during COVID-19 related to personal circumstances and wellbeing, assessing and managing risk, relationship issues, financial and regulatory processes, and virtual engagement.

### Personal circumstances and wellbeing

Participants described how researchers and partners were busy, stretched thin, and stressed during the pandemic. Many team members were essential workers and overburdened with increasing demands they faced in managing the COVID-related health crisis. Care demands also increased, as schools and daycares closed, and team members became responsible for caring for their children while working. Overall, people were tired and overworked during this time and navigating many competing priorities, making it challenging to sustain ongoing CEnR efforts.

*Suddenly, I went from having full-time childcare to having all […] of my children at home and having to facilitate online schooling […] while still continuing to teach my classes and do my work […] I had people come to me […] and say things like, “Oh, there’s all these great opportunities. There’s so much money to do this work right now because of the pandemic. Have you applied for any of these? Are you looking at these?” I said, “I’m in no position right now […] with […] children at home, a full-time job, no care […] to be doing any of that.” (Researcher 2)*

*I think that researchers were so overloaded with whatever kinds of things […] they had to change because of COVID demands. All the ones that were working with new COVID patients, a lot of them I think were just unable to dedicate time to projects that were moving along pretty nicely before COVID started. (Partner 1)*

People also experienced personal hardship, including sickness, losing family members, dealing with inequities exacerbated by COVID-19, and experiencing trauma related to COVID-19. With these hardships taking precedence, people were unable to focus on research.

*Community members not only were more difficult to reach through electronic means, they were also really struggling with the inequities that COVID-19 exacerbated. People didn’t have stable housing, they may or may not have had access to internet. They were definitely focused on survival and not on participating in a research network. (Researcher 5)*

*I think that sometimes researchers feel like we’re not giving 100 percent and showing up, but I know for myself that things just keep coming up. They just seem to be cropping up out of nowhere. Someone’s sick, or an accident has happened. Life stuff has really gotten in the way. It’s not that we’re making excuses. (Partner 8)*

A couple of researchers described receiving support requests they were not fully prepared to handle or dealing with increased emotional labor while in a state of hardship.

*COVID increased the amount of social support that we as researchers needed to provide to our community partners […] We did meetings late at night, and they were longer. I got more text messages and more phone calls, and more distress. […] With COVID, I was being affected, too. Normally, you’re in a bit of a place where you can deliver that support, because we’re lucky, right? We have jobs and homes, and we’re okay. For the most part, we’re okay. We weren’t okay during COVID. (Researcher 7)*

### Assessing and managing risk

Participants who had planned to conduct in-person activities highlighted challenges associated with assessing and managing COVID-19 risk, with some expressing concerns about putting themselves and others at risk of infection through continuing their work; this issue affected local projects specifically. One researcher shared how this was especially a concern when working with vulnerable populations who had fewer resources to navigate illness:

*The people we work with serve high-risk, low-income women who have children that are under five years of age and may be pregnant. It would be too easy to expose one of them. I just had to make an ethical call that even if I had thought the partnership wouldn’t have survived that, I couldn’t send a student there. I couldn’t take a risk of exposing one of our participants or one of our moms who’s a board member. (Researcher 2)*

### Relationship issues

Some researchers and partners, particularly those with local partnerships, described challenges with team relationships during the pandemic. For instance, teams were not able to rely as much on in-person interactions, which hindered the relationship-building process for some.

*My plan was to travel the state, you know, do the Southern thing, where people do much better face-to-face, maybe have a meal together and just sort of set up a plan to do that […] Then none of that was possible. (Researcher 5)*

Partners described how the principles of community engagement were compromised when they were not able to meet in-person, leading to less collaboration, people being more guarded with their time, and less negotiation to ensure mutual benefits between researchers and partners. One partner shared:

*With COVID, I feel like it pushed us backwards. If there’s a piece that has to be done at the University now, it’s done at the University […] There is no tradeoff in terms of the work. The researchers, of course, have to make sure that their work gets done. They do whatever they have to do; whereas, before it was more of a give-and-take type of situation. (Partner 8)*

This led this partner to become less engaged in the work, only taking on projects where she felt less “*skin in the game*,” rather than projects affecting her community. Similarly, a few researchers felt personally affected by decreased in-person relationship-building:

*I need to be in the community. I wanna meet people. I wanna see it in action. That’s why I do what I do. I feel like it affected me as a researcher big time […] I got depressed being on Zoom meetings all day long and feeling overwhelmed and all the additional things we had to deal with as researchers. (Researcher 7)*

Relationship issues were not as pervasive for participants who engaged in non-local projects. However, some working on regional or national level projects still described challenges with building new partnerships during the pandemic, especially in cases where an initial in-person event would have been planned under normal circumstances.

*I’ve never met any of these groups […] In a natural environment, in normal circumstances, the [regional] coalition would’ve met in person. We would’ve met as team members […] It’s been very, very different. (Partner 5)*

### Financial & regulatory issues

Several participants discussed funding-related concerns and challenges during the pandemic. These included not being able to fundraise as they did before, challenges processing finances quickly, supply chain issues, increasing costs of supplies, and fewer work opportunities for partners.

*We have had some challenges due to COVID […] the work not being as plentiful […] I do feel like we would’ve been further along on some projects or even writing some grants and stuff had COVID not came in […] it slowed it down a lot. I think there are definitely financial implications of it, but I’m hopeful that we’ll rebound from that. (Partner 8)*

Most of the financial challenges researchers discussed were not related to COVID-19 specifically (e.g., regulatory or funder limitations that made it difficult to compensate partners) but were perceived as even greater hurdles to overcome in the pandemic context. One researcher expressed concerns about meeting funder’s requirements when experiencing project delays during COVID-19.

Additionally, several participants discussed IRBs, supervisors, or other governing bodies not allowing or restricting in-person engagement, leading to delays and struggles to find workarounds. One partner discussed the burden of increased pandemic-related paperwork, as well as the challenges imposed by rules around in-person work, such as not having access to bathrooms, and concerns about consequences if rules were broken:

*I mean, literally, if you broke the rules, your project could’ve been closed. You followed the rules. Plus, who wants to take a chance that you don’t follow the rules, and someone gets COVID and it’s your fault? No one wants to do that. I would say that a difference would be, things became much more formalized, and the interactions were limited. (Partner 2)*

### Virtual engagement

We conceptualized virtual engagement as remote interactions using an electronic platform, including phone, email, shared online documents, and videoconference platforms. Given the many virtual engagement challenges noted by participants, these are displayed in table format (Table 2).

**Table 2.** Virtual engagement challenges

Challenge	Examples	Example Quote
Difficulty building relationships in a virtual space	Harder to network and make connections People being more guarded in their speech Fewer personal interactions, less small talk Difficult to read tones and body language	*[In email or Zoom meetings] I don’t get to ask the questions. I don’t get to say, “How is little Johnny,” or “How’s the soccer game?” There’s no more personal interactions anymore. It’s more just about the business, which makes it seem so, so, so professional and not anywhere near where we used to be. (Partner 8)*
Difficulty understanding community context	Harder to understand the context of a particular site or community when working virtually (especially challenging for teams with local partners previously relying on in-person interaction)	*We had talked a little bit about, at some point, them [research team] being able to come down and meet with people, and that never was able to happen […] Again, if you’re not the one out in the trenches, there is some stuff that you don’t understand about the work ’cause it’s different. (Partner 3)*
Difficulty maintaining connections and focus during virtual interactions	Multitasking while in meetings Experiencing distractions outside of the screen Virtual engagement fatigue (e.g., difficulty staying in long meetings, too many emails) People stop responding to digital outreach	*We did a lot of advisory board meetings with screaming babies and toddlers running around and pulling stuff down and ripping phones out of Mom’s hand. It just complicated everything. (Researcher 2)*
Logistical issues	Hard to schedule and get people to attend virtual meetings Conflicting priorities Meetings feeling rushed No longer able to rely on quick in-person interactions as a meeting alternative	*I think people had competing priorities because there were so many Zoom meetings and because you had to have a Zoom for practically everything you did, because you could not see each other. You weren’t in the office together, so you could not have a quick meeting that way, so everything required a schedule. You had to schedule all of your interactions with your colleagues and peers. (Partner 1)*
Accessibility and functionality of technology	Lack of access to quality Internet, affordable data plans, or devices like computers Technology failures or Internet glitches Learning curve/time required for learning about and becoming comfortable with new platforms/technologies	*Early on, having a strong enough internet connection to be present all the time [was a challenge], so I ended up having to get a second router—a booster so that I would have a strong enough connection that I didn’t lose my signal. I think that was huge. I think working with families who live in rural spaces where internet is sketchy—so being able to use all of these applications and platforms is wonderful, but if you do not have the technology bandwidth to do it, then you’re still in the desert. (Partner 6)*

### Non-COVID or non-CEnR-related challenges

While not this project’s focus, many participants described engagement challenges not related to COVID-19 specifically, but that affect CEnR broadly. These included challenges compensating partners, language barriers, communication issues, negative perceptions of research or the university within the community, and partners working with limited resources. In addition, many participants discussed challenges teams faced during COVID-19 related to study implementation, such as restrictions on in-person study activities leading to delays or requiring project changes, difficulties navigating regulatory requirements under new circumstances, and challenges collecting data or obtaining consent virtually.

### Facilitators

Participants discussed existing resources, strengths, or circumstances carried into the COVID-19 pandemic that facilitated CEnR, as well as supports that were available or beneficial to their team during this time, which we are calling “foundations.” They also discussed strategies and practices developed in response to a challenge or issue and used to support engagement.

### Foundations

#### Partnership factors

Participants identified the expertise and lived experience of partners as a key foundational factor in supporting CEnR during COVID-19. Partners leveraged their lived experiences and community expertise to help research partnerships and projects navigate the unique pandemic context.

*[Community partners] know the context better than we do. They know the settings. They know the participants. We listen to them, and they know we listen to them because of how we engage with each other. Experiential knowledge is just as important as the research side of things. (Researcher 9)*

Additionally, researchers and partners with strong relationships from previous collaboration – whether at the local or national level – described how shared understanding, trust, mutual respect, and knowing each other personally helped them weather challenges and maintain their partnerships through the pandemic.

*I think there are a lot of things that have fallen out as a result of COVID that were unintended impacts. The good thing is, though, when you have a strong partnership, it doesn’t matter how you don’t see each other. When you do see each other, it’s like you’ve never been apart. (Partner 8)*

#### Funding & institutional support

Multi-year funding periods and less stringent funding mechanisms (e.g., departmental or institutional funding, funding through non-federal sources) facilitated researchers’ ability to consistently compensate and engage partners. Researchers also mentioned that it was helpful working with funders who did not strictly enforce administrative obligations during the pandemic, allowed flexibility to pivot funding to other activities, and provided access to additional human resources such as an engagement officer.

*It’s a foundation. I think they were wrestling with the same things at the same time in doing their own work. They were willing to experiment along with us, and learn along with us, because they were shifting a lot of their own programming to virtual environments. I think we just approached it as a shared learning experience. (Researcher 10)*

For some researchers, support from institutional leadership – such as allowing for more lenience in administrative processes (e.g., accepting electronic signatures), giving employees access to virtual platforms, and offering free supplies like masks and hand sanitizer – was beneficial.

#### Project-specific factors

Certain project-specific factors – such as geographic scope, previous engagement strategy, and design – facilitated engagement throughout the pandemic. Participants shared how CEnR partnerships that were not local (e.g., regional or national) and were already meeting virtually prior to COVID-19 were not as adversely impacted. Instead, the pandemic presented some silver linings, with evolutions in and proliferation of virtual engagement platforms strengthening collaboration, relationships, and engagement.

*We’re based remotely, we do not have office space. Our internal teams do not meet in person. We didn’t have to ramp up with how are we going to do it, the structure’s already there, so then just focusing on, number one, identifying what are the new sets of needs for the patients, and how do we meet those creatively in a virtual environment? (Partner 4)*

### Strategies

#### Communication and flexibility

Participants highlighted the importance of transparent communication, especially in regard to respecting boundaries and being honest about limited capacity, competing demands, and wellbeing. Researchers in the early phases of their CEnR projects also discussed the importance of being intentional about which community members to approach for partnership, given these challenges.

*I would say that we had to be really honest about what we were capable of. Like, emotionally, financially, physically. What could we really do that honored the spirit of the project, but also honored our own limited capacity, given everything that was going on. (Researcher 5)*

Approaching CEnR with increased flexibility and creativity was also a strategy that allowed participants to adapt and pivot their work and be open to new project directions. Some researchers mentioned offering services, resources, and support to partners to help maintain trust during the pandemic.

*We collected funds for our community partners to buy masks and things […] There was a lot of pivoting to just helping people connect to food resources in their community, to PPE, to protection equipment, masks, a lot of that. Some of our projects where we had to stop, we did a lot more basic needs work. (Researcher 1)*

#### Risk mitigation

While most participants pivoted to virtual engagement during the pandemic, those who continued in-person activities discussed strategies for mitigating risk of COVID-19 transmission during in-person interactions, including wearing masks, disinfecting surfaces, meeting outdoors, social distancing, and implementing vaccination and testing requirements. For some participants with local partnerships, determining how to have in-person interactions was an important strategy to maintain CEnR projects and relationships with partners.

*We met outdoors with masks. It was more of a one-on-one or one on three or four kind of thing, rather than having a bunch of people in a room. (Partner 2)*

#### Virtual engagement

Finally, many participants discussed strategies they used to address challenges related to virtual engagement. Given the many concrete strategies cited, these are displayed in table format (Table 3).

**Table 3.** Virtual engagement strategies

Type of Strategy	Examples	Example Quote
Supporting use of technology	Orienting partners to new virtual platforms (e.g., one-on-one meetings or practice sessions) Texting to communicate with partners with limited data plans or Internet access	*We do a lot of texting, actually, with the [partners] who joined our board. I know it’s ‘cause their text messages are free on many of those plans. (Researcher 2)*
Utilizing tools and features in virtual platforms to enhance engagement and accessibility	Breakout rooms to encourage small group discussion Screen sharing documents to receive feedback in real time Live polling Accessibility features such as alternative text, closed captioning, and text description of images Spanish and American Sign Language interpretation for large virtual convenings	*Just to make sure that we weren’t just assuming everybody could see whatever was on a visual, but talking through all of the things, creating Alt Text. Closed captioning as well for people who might not be able to hear. That was really helpful to us. (Researcher 5)*
Setting expectations & prioritizing discussion items	Ground rules at the start of virtual meetings (e.g., remain on mute when you are not speaking, use the raise hand function, etc.) Sending and asking individuals to review meeting materials and agendas in advance	*We try to set ground rules at the beginning. If you’re on the call, turn on your video. Use the raise your hand thing […] planning for what the goals are of the given meeting […] Following up with action items afterwards. (Researcher 8)*
Flexibility in scheduling virtual meetings	Having multiple meetings covering the same content to accommodate people in different time zones Rescheduling meetings outside of business hours	*To meet the needs of the team, whether that was a 6 pm meeting as opposed to 9am […] I can remember throughout the ages, it used to be “Oh they can’t come. Let’s meet anyway because we need to move forward.” Instead of doing that it was, “Well, we need the whole team here. Let’s find another time.” (Partner 6)*
Humanizing virtual interactions	Starting meetings with icebreakers Building in time for informal conversation Flexibility in virtual interactions (e.g., allow breaks, cameras off, use of chat) Using chat to reach out to people one-on-one	*What I do is when I’m on Zoom, I’ll take the opportunity to send a private chat to say, “Hey, missing you,” or, “Hey, we haven’t connected. Hope all is well,” or something like that. (Partner 8)*

Notably, many participants shared that shifting to remote work facilitated positive changes to collaboration, workflow, and relationship dynamics. Virtual engagement provided opportunities for people in different geographic locations to work together, made engagement more accessible, and offered glimpses into others’ lives.

*You’re often getting a window into people’s homes or people’s work environments. Seeing people’s family members in the background […] or their dog sticks a head into the Zoom. I think if you’re prepared to accept those things, and be open to them, then they can be pretty humanizing and flattening in a good way for a group of mixed backgrounds. I wouldn’t say it totally neutralizes whatever existing power dynamics there are, but it certainly flattens some of them. (Researcher 10)*

Furthermore, some partners expressed that fewer in-person meetings created more time to be creative and pursue other engagement ideas or introduced flexibility to use funds for other things, like expanded programming.

#### Desired supports

Participants described several suggested or desired supports for CEnR during future health emergencies like the COVID-19 pandemic, including recommendations related to funding, virtual engagement guidance and accessibility, and tips for navigating CEnR during crises. While many described desired supports to facilitate CEnR generally (e.g., improved community outreach and communication, training for researchers on the benefits and process of engagement), supports that were specifically related to emergent conditions are listed in Table 4.

**Table 4.** Desired supports

Category	Desired Supports	Example Quote
Funding	Greater flexibility from funders during crises like the pandemic Greater flexibility for use of funds (e.g., being able to purchase gift cards for food during virtual meetings) Financial support for partners during difficult times	*I think with the other [funder], a little more flexibility with that, feeling like if we didn’t find a way to spend out this money and make it work, despite the pandemic, that we could potentially lose it and not be able to complete the project at all would have been helpful. (Researcher 2)*
Guidance about navigating research during crises	Guidance on navigating engaged research during crises like the pandemic Contingency plans Roadmap for switching from an in-person to a virtual study [not engagement-specific]	*If everyone who’s done a project during COVID could put together a how-to manual on how to engage your end-user, your stakeholders, in the process and give examples […] I think we learned some things in COVID—all of the projects who continue to do research that can be shared amongst all of us, both the researchers and the community engagement teams. (Partner 6)*
Guidance about virtual engagement	Resources for navigating hybrid environments Tips/guidance for virtual engagement/facilitation Tips on addressing accessibility issues in virtual engagement Guidance on other virtual platforms that certain populations (e.g., youth) may prefer Guidance on how to show appreciation to partners when engaging virtually	*This is a hybrid environment. We’re all living in it now. I think people are thinking about it now and I hope that there’ll be more resources developed in the future about, just in general, how do you work and live in a hybrid environment? (Partner 2)* *I think really practical tips or resources about these Zoom meetings where you’re bringing a lot of people together, any kind of tried-and-true tips […] I think we know now, you can do breakout rooms. You can do a poll, but maybe it would be helpful to kind of have all of that in one place, and if there are other functions that people aren’t thinking about with Zoom. I think that would be helpful. (Researcher 3)*
Virtual engagement accessibility	Improved access to Internet and communication tools, including broadband access in rural areas Access to Zoom & other Webinar platforms provided to partners Trainings on using Zoom or Teams for researchers and partners, including trainings offered in multiple languages	*We need rural broadband. We need wide coverage of Wi-Fi in rural communities. (Researcher 1)* *Provide peer counselor types, those of us who are out in the community, who are the bridge between the researchers […] We need more people in the community that are trained […] in how to use Zoom or WebEx meetings and have it in their language. (Partner 3)*

## Discussion

The COVID-19 pandemic brought about many changes to the research enterprise, including studies using CEnR approaches. We sought to better understand researchers’ experiences engaging partners in health-related research during COVID-19, as well as partners’ experiences engaging in research during this time. Researchers and partners faced many challenges affecting their ability to work together and maintain partnerships during COVID-19. At the same time, the strengths and resources that teams carried into the pandemic and the strategies they developed to address challenges allowed teams to persevere.

Experiences of both researchers and partners varied based on project- or partnership-related factors. We observed that CEnR projects that were national in scope reported fewer impacts of COVID-related challenges, in part because they were already accustomed to conducting work virtually. In fact, some involved in these types of projects reported positive changes to their workflow and relationship dynamics due to improvements in and proliferation of videoconferencing platforms, allowing for “face to face” interactions (as opposed to conference calls and emails). Alternatively, teams with local partnerships that engaged community members through a community-based participatory (CBPR) approach [1] or previously relied on in-person engagement reported greater disruptions to their work and relationships. Researchers also described differences in CEnR experiences based on the types of collaborating partners (e.g., patients, community members, healthcare providers). For example, projects that engaged providers faced unique difficulties, as providers had to navigate the challenges of responding to pandemic-related healthcare needs. Our findings suggest that higher-level engagement (e.g., CBPR) might be more negatively impacted during crises than lower-level engagement. Therefore, institutions should allocate resources appropriately, such as providing additional funding and guidance to projects with higher-level engagement goals. CEnR teams may also benefit from resources that offer tailored strategies for navigating future health emergencies depending on engagement methodology and the types of partners engaged, as well as guidance on how community engagement principles [25] can be maintained and strengthened when in-person collaboration is not feasible.

While COVID-19 presented a unique set of challenges for CEnR teams, specifically around personal circumstances and wellbeing, assessing and managing risk, and virtual engagement, many participants described barriers that have long affected CEnR projects [26–28]. Many challenges described by our participants – including financial and regulatory barriers – reflect institutional-level policies, processes, and structures that do not support CEnR and negatively impact partnerships and projects, as reported in other studies [27,29,30]. Ours and other studies have shown how these challenges were amplified and exacerbated in the context of COVID-19 [31–34]. In addition to addressing the unique challenges that arose during COVID-19, our findings emphasize the importance of continuing to address these long-standing barriers to CEnR that often fall beyond the control of individual researchers [35–38]. While researchers can be strong advocates for systems-level change that supports CEnR at their institutions, such as by communicating with senior leadership about the value of CEnR and building relationships with key regulatory and administrative departments whose policies impact CEnR [39], the time, effort, and flexibility needed to do this is often not built into funding and research agendas or encouraged within traditional promotion and tenure policies at academic research institutions [35,40,41].

Furthermore, researchers focusing on projects unrelated to COVID-19 had to deal with funding drawbacks and resource constraints, all while navigating numerous personal challenges brought about by COVID-19 [42]. As observed in our study, researchers spoke in candid and humanizing ways about how the pandemic affected their mental, emotional, and physical wellbeing. Some researchers discussed challenges of balancing family caretaking with research responsibilities while receiving little institutional support or flexibility. While some research has been done around training and professional development needs within the clinical and translational science workforce prior to the pandemic [43], further research is needed to explore wellbeing within the CEnR workforce [14].

The pandemic has created a new landscape for CEnR and partner collaboration, and there will likely be future crises affecting CEnR partnerships. Funders can play an important role in ensuring that CEnR is supported during crises by offering more flexibility in the ways funds can be used to support partners, adapting contingency plans that are ready to implement in times of disruption, funding learning networks to share best practices for navigating CEnR during health emergencies, and offering resources to strengthen virtual or hybrid CEnR [29,34,44]. Notably, our participants’ desired supports touched upon the importance of addressing structural barriers related to digital literacy, access to Internet, and access to basic material resources (e.g., electricity, protective equipment, etc.) that affected researchers’ and partners’ ability to continue their work together [45]. Aligning research priorities and outcomes with tangible benefits for communities may facilitate more successful continuation and greater impact of CEnR projects during future crises, and could help establish researchers and their institutions as more trustworthy partners in CEnR [46,47].

Our study has several limitations. Due to our purposive and snowball sampling approach limited to CEnR projects affiliated with only our academic research institution, further investigation is needed to determine how applicable our findings are to other settings. Additionally, findings might differ in a more diverse group of participants, such as in CEnR conducted in languages other than English. There was some confusion amongst participants about differences between research participation and research engagement, leading to some conflation of experiences with research implementation and research engagement. Perhaps this could have been addressed by providing a more detailed definition of CEnR and probing more on responses to disentangle the nuances between study implementation and engagement, though in our analysis we did take care to focus on engagement experiences. Finally, while we note nuances in CEnR experiences among projects with different geographic scopes (e.g., local vs. national) throughout this paper, this theme emerged during analysis and was not included in our initial research aims or questions. As such, future research could explore this topic further.

Despite these limitations, our study offers unique insight into researchers’ and partners’ experiences and perspectives of research engagement during COVID-19. While other publications have discussed virtual engagement approaches [48–52] and experiences of research engagement broadly [53–60], they have largely focused on a single health condition or project, were conducted in other countries, collected data quantitatively, focused only on researchers’ perspectives, and have been shared via case studies, perspective pieces, or methods papers. This paper contributes to the limited body of literature related to US-based qualitative research on this topic and explores perspectives of both researchers and partners across a range of projects focused on varying health conditions, locations, and engagement methods. Importantly, despite these differences, our findings reflect some of those shared in the aforementioned studies (e.g., challenges, strategies, and recommendations related to virtual engagement; impacts of COVID-19 on CEnR partnerships, etc.), thus highlighting the potential transferability of our findings to other settings and contexts. Furthermore, our study elaborates on findings that were not extensively discussed in other publications, such as financial and regulatory issues, funding and institutional supports, and project-specific factors like geographic scope and level of engagement. Based on our study’s unique findings, we have included a list of key recommendations (Table 5) that could be considered alongside more comprehensive proposed playbooks for responding to future epidemic public health challenges [16].

**Table 5.** Key recommendations from the authors for conducting effective CEnR during public health crises, based on study findings

**Recommendations for Funders:** Allow for flexibility in project timelines, reporting requirements, activities, deliverables, and how funds may be spent instead of strictly enforcing administrative obligations. Create emergency pools of funding and provide subsidies for CEnR partnerships to access when implementing projects during crises. Organize synchronous (e.g., live panels, webinars) and asynchronous (e.g., online discussion boards) forums to share information with grantees about how to adhere to funder rules and regulations. Prioritize providing funding and guidance to CEnR projects with higher-touch engagement goals (e.g., CBPR projects) and/or local CEnR projects, as these types of projects may be most negatively impacted during crises.
**Recommendations for Institutions:** Communicate transparently about if, how, and why institutional priorities may shift during crises. Acknowledge the mental strain that crises pose for research team members and offer support, such as directing individuals to mental health resources, encouraging flexibility in working modalities, or allowing additional time off from work to cope with challenging/distressing experiences. Proactively develop and disseminate guidelines about operationalizing CEnR activities during crises that describe changes researchers may utilize to minimize risk for in-person engagement or shift to virtual activities. Provide institutional funding and resources to support community engagement (e.g., funding for community partner compensation, access to online platforms and videoconference software).
**Recommendations for Research Teams:** Seek to build strong relationships with community partners prior to times of crisis. Proactively develop project-specific emergency preparedness plans that may be implemented during crises. Communicate with community partners about topics such as capacity for engagement and how project and engagement activities may be impacted by a crisis; consider developing a written agreement of collaboration. Utilize strategies to enhance virtual engagement (see Table 3).

CEnR, community-engaged research; CBPR, community-based participatory research.

COVID-19 emphasized the importance of integrating community voice and perspective in research [19], leading to renewed calls for making community engagement an essential part of all clinical and translational research. To heed these calls to action, it is important to explore the experiences of those who conducted CEnR during COVID-19 to better understand the contextual challenges and facilitators they experienced. This study provides important insights into the experiences of researchers and partners conducting CEnR during the COVID-19 pandemic. Our findings can be used to help create resources and strategies to support CEnR partnerships during future challenging times.

## Supporting information

10.1017/cts.2025.10090.sm001 Frank et al. supplementary material 1 Frank et al. supplementary material

10.1017/cts.2025.10090.sm002 Frank et al. supplementary material 2 Frank et al. supplementary material

## Supplementary material

The supplementary material for this article can be found at https://doi.org/10.1017/cts.2025.10090
