# Supplementary material for: Researcher and community partner perspectives on community-engaged research during the COVID-19 pandemic
Source: J Clin Transl Sci. 2025 Jul 7;9(1):e163. doi: 10.1017/cts.2025.10090 (PMC12392356; doi:10.1017/cts.2025.10090)
Supplement: Frank et al. supplementary material 1 — Frank et al. supplementary material [file S2059866125100903sup001.pdf]

Note: These are semi-structured interviews. The questions below will guide the interviews but may vary based on the conversation.

Contents

Researcher Interview Guide....1
Stakeholder Partner Interview Guide....3

Researcher Interview Guide

To begin, I'd like to learn about your general experience working on stakeholder-engaged health-related research projects. We consider stakeholder-engaged research projects to be those that meaningfully involve stakeholders from outside the university in the design and/or conduct of a research study at any stage of the project – from planning the study, to conducting the study, to disseminating study information or results. Engagement can be everything from stakeholders providing feedback on a study, to stakeholders being investigators on the study. We consider stakeholders to be anyone outside of the academic research setting who are or may be impacted by the conduct or outcomes of the research (e.g., people who work at community organizations, patient advisors, healthcare workers).

1) First, please tell me about your experience working on stakeholder-engaged research projects **before** the COVID-19 pandemic began. Probes:
    a. How long have you been working on these projects?
    b. What has been your role?
    c. What types of stakeholders have you engaged with?
    d. How have you engaged with stakeholder partners?

Thank you for sharing that background information! Now I'd like to hear about your experience ***during the COVID-19 pandemic, or as of March 2020****.*

2) Tell me about the stakeholder-engaged research projects you've worked on during the COVID-19 pandemic. Probes:
    a. What were the purposes of these projects?
    b. What has been your role in these projects?
    c. What types of stakeholders have you engaged with?
    d. How have you engaged with stakeholder partners?
    e. Have these projects started during the pandemic, or continued from before the pandemic?

3) What has it been like for you and your stakeholders to work together on these projects during COVID-19? Probes:
    a. How was your relationship impacted by the pandemic?
    b. What were some of your stakeholder's experiences engaging in research during the pandemic? How did you find out about these experiences?

4) What strategies have worked well when engaging with stakeholders on research projects during the COVID-19 pandemic? Probes:
    a. *If not mentioned:* What strategies, if any, have worked well related to virtual or non face to face (ie: telephone) engagement?

5) What challenges have you or your team encountered when engaging with stakeholders on research projects during the COVID-19 pandemic? Probes:
    a. *If not mentioned:* What challenges, if any, have you experienced related to:
        i. Virtual or non face to face engagement?
        ii. IRB or other regulatory issues?
        iii. Financial management or compensation?
    b. How have these challenges affected your work?
    c. How, if at all, have you addressed these challenges?
    d. Were there any research activities that couldn't continue because of COVID? If so, what were the reasons you could not continue? What would have helped you continue?

6) Considering your experiences so far, what resources or training might help you or other researchers more effectively engage with stakeholder partners moving forward during the COVID-19 pandemic or other health emergencies? [if this does not come up spontaneously, probe around]:
    a. What resources and trainings might be specifically helpful for virtual or non face-to-face engagement?

7) Thank you so much for sharing your perspectives today! Before we end, I have a few demographic questions, which we are asking all interview participants:
    a. What is your gender identity?
    b. What is your race?
    c. Are you Hispanic or Latino?
    d. How many years have you worked in your current position?
    e. How many years have you worked with stakeholder-engaged research projects?

8) [If relevant] Lastly, I was wondering if you might be able to help us connect with additional people who might be interested in participating in this study.
    a. We would greatly appreciate it if you would share this information with some of your stakeholder partners or other researchers who have worked on health-related research studies at UNC-Chapel Hill during the COVID-19 pandemic. Would you be willing to forward the information in my original email to stakeholders, researchers, or research staff who might be interested in participating? I would be happy to re-send that email if helpful. Stakeholder partners will be offered a $50 gift card for participating. If they are interested in participating, they may contact me at [name/email].

Stakeholder Partner Interview Guide

To begin, I'd like to learn about your general experience working on stakeholder-engaged health-related research projects with academic researchers. We consider stakeholder-engaged research projects to be those that meaningfully involve stakeholders from outside the university in the design and/or conduct of a research study at any stage of the project – from planning the study, to conducting the study, to disseminating study information or results. Engagement can be everything from stakeholders providing feedback on a study, to stakeholders being investigators on the study. We consider stakeholders to be anyone outside of the academic research setting who are or may be impacted by the conduct or outcomes of the research (e.g., people who work at community organizations*, patient advisors, healthcare workers).*

1) First, please tell me about your experience working on research projects with academic researchers before the COVID-19 pandemic began. Probes:
   a. How long have you been working on these projects?
   b. What has been your role on these projects?
   c. What types of researchers have you engaged with?

Thank you for sharing that background information! Now I'd like to hear about your experiences ***during the COVID-19 pandemic, or as of March 2020.***

2) Tell me about the health-related research projects you've worked on with academic researchers during the COVID-19 pandemic. Probes:
   a. What has been your role?
   b. What types of researchers have you engaged with?
   c. Have these projects started during the pandemic, or continued from before the pandemic?

3) What has it been like for you and your researcher partners to work together on these projects during COVID-19? Probes?
   a. How was your relationship impacted by the pandemic?
   b. During the pandemic, what was something you learned about your researcher partners' experiences that affects their ability to engage stakeholders in research? How did you become aware of it?

4) What, if anything, has helped you feel supported when working with researchers on projects during the COVID-19 pandemic? Probes:
   a. *If not mentioned:* What, if anything, has helped you feel supported when working in virtual or non face to face settings, like Zoom?

5) What challenges have you or other stakeholders encountered when working with academic researchers on research projects during the COVID-19 pandemic? Probes:
   a. *If not mentioned:* What challenges, if any, have you experienced related to:
      i. Working with researchers in virtual or non face to face settings, like Zoom?
      ii. Meeting IRB or other regulatory requirements?
      iii. Being compensated for your work, or other finances
   b. How have these challenges affected your work?
   c. How, if at all, have you or your researcher partners addressed these challenges?
   d. Were there any research activities that couldn't continue because of COVID? If so, what were the reasons you could not continue? What would have helped you continue?

6) Considering your experiences so far, what resources or training might help you or other stakeholders more effectively work on research studies with academic researchers moving forward during the COVID-19 pandemic or other health emergencies? [if this does not come up spontaneously, probe around]
    a. What resources and trainings might be specifically helpful for virtual or non face-to-face engagement?

7) Thank you so much for sharing your perspectives today! Before we end, I have a few demographic questions, which we are asking all interview participants:
    a. What is your gender identity?
    b. What is your race?
    c. Are you Hispanic or Latino?
    d. How many years have you worked on research studies with academic researchers?

8) [If relevant] Lastly, I was wondering if you might be able to help us connect with additional people who might be interested in participating in this study.
    a. We would greatly appreciate it if you would share this information with other stakeholder partners or researchers who have worked on health-related research studies at UNC-Chapel Hill during the COVID-19 pandemic. Would you be willing to forward the information in my original email to stakeholders, researchers, or research staff who might be interested in participating? I would be happy to re-send that email if helpful. Stakeholder partners will be offered a $50 gift card for participating. If they are interested in participating, they may contact me at [name/email].
