# Supplementary material for: Researcher and community partner perspectives on community-engaged research during the COVID-19 pandemic
Source: J Clin Transl Sci. 2025 Jul 7;9(1):e163. doi: 10.1017/cts.2025.10090 (PMC12392356; doi:10.1017/cts.2025.10090)
Supplement: Frank et al. supplementary material 2 — Frank et al. supplementary material [file S2059866125100903sup002.pdf]

Code	Type	Description	Example
**Accessibility**	Memoing	Apply this code when participants discuss accessibility as it is related to inequity or equity (e.g., interpretation, closed captioning, internet access), related to research project or partnership.	"One of the goals of our project is to reach mothers who are surviving on low incomes and surviving poverty. That puts a barrier into doing work over Zoom remotely because many of them can’t afford good internet access if it’s available in their area." [Particpant ID Redacted]
**Assessing risk**	Added during coding	Apply this code when participants discuss taking into account potential for adverse outcomes related to COVID-19 when making decisions related to their projects.	"Also, the fact that a lot of our, the people we work with serve high-risk, low-income women who have children that are under five years of age and may be pregnant. They can’t even have their children vaccinated yet. Even though the risk is lower in children, there’s no protection if we go. It would be too easy to expose one of them." [Participant ID Redacted]
**Challenges / Barriers**	Pre-determined (guide)	Apply this code when participants discuss challenges or barriers affecting engagement of research team, stakeholder partners, or participants in the research partnership or study.	"These are our main agency partnerships. When the pandemic hit, because they were all essential workers, they couldn’t just start working remote like I did. Not only was it that we couldn’t be there in person with them, it was that they had so much more on their plate that we didn’t feel like we could ask anything of them. Things really did just kind of grind to a halt, initially." [Participant ID Redacted]
**Desired resources**	Pre-determined (guide)	Apply this code when participants discuss desired or suggested resources or training to support engagement.	"...a little more flexibility with that, feeling like if we didn’t find a way to spend out this money and make it work, despite the pandemic, that we could potentially lose it and not be able to complete the project at all would have been helpful." [Participant ID Redacted]

**Facilitators - Foundations**	Memoing	Apply this code when participants discuss built-in or existing resources, strengths, or circumstances carried into the pandemic (e.g., existence of a relationship before COVID) that facilitate or support engagement (or the project).	"I don't know if I would have been able to manage and sustain the partnership if it had not been very strong before the pandemic. I think because it was, I've been able to maintain it." [Participant ID Redacted]
**Facilitators - Other**	Memoing	Apply this code when participants discuss factors that support or facilitate engagement that are not foundations or strategies (e.g., funder was flexible)	"One saving grace of that project was how flexible the funders were and how we didn't really have all of these administrative reporting obligations to them." [Participant ID Redacted]
**Facilitators - Strategies**	Pre-determined (guide)	Apply this code when participants discuss experiences or perceptions around strategies or practices used to facilitate/support engagement. Strategies are reactive to a challenge or issue.	"It's a community where a lot of people come and go, and they expect that. I think the fact that I keep—I hope the fact that I keep showing up shows that I'm not going to go anywhere." [Participant ID Redacted]
**Financial - compensation**	Added during coding	Apply this code when participants discuss, perceptions, issues or practices related to compensating stakeholders.	"But of course, these things are hard to compensate people for. They're not full-time jobs, so you're working with that aspect. They've been very active and very supportive. I try to give back to them where I can." [Participant ID Redacted]
**Financial - funder/ing**	Added during coding	Apply this code when participants discuss perceptions, issues, or practices related to research/er funding and funders.	"Fortunately, the little pots of money that I had were not federal dollars, so they don't come with all of the regulations. The places where I had that funding from were able to be more flexible and more generous with it." [Participant ID Redacted]

**Financial - other**	Added during coding	Apply this code when participants discuss financial issues other than compensation or funder/ing.	"Well it all had to keep moving. Right?...we had to do the rain barrels. They were hard to find, but eventually we found some. Of course, the prices were doubled, and I'm like 'Oh no'". [Participant ID Redacted]
**Impact on relationships**	Pre-determined (guide)	Apply this code when participants discuss impact (or lack of impact) of COVID-19 related circumstances on interpersonal/relationship dynamics/experiences (outcomes, feelings, etc.) between researchers and stakeholders or participants.	"It's interesting because it's one of the most diverse advisory boards I've ever worked with. That was purposeful because of our diversity, equity, and inclusion mission in our center. But it just wasn't impacted as much. It was all meant to be remote and not as participatory." [Participant ID Redacted]
**Impact on work**	Memoing	Apply this code when interviewee discusses impact (or lack of impact) of COVID-19 related circumstances on their research, work, project, etc. outcomes.	"We were in the process of planning a community forum as the culminating event of the Photovoice project. It was supposed to happen in May of 2020. Was it April or May? I can't recall. Time has moved so oddly during the pandemic. It was supposed to happen then, and we had to postpone it and postpone it." [Participant ID Redacted]
**New insights**	Added during coding	Apply this code when participants discuss learning new things about themselves, their work, or their stakeholder partners throughout the COVID-19 pandemic.	"I knew that they had experienced natural disasters before. I knew that it had been traumatic for the community...I work with individuals who experience trauma quite a bit. I know how trauma can impact a family, but to see that flip, like a light switch in an entire group. Just the whole dynamic of the board that first time we talked to them was so different." [Participant ID Redacted]

**Persevering**	Added during coding	Apply this code when participants discuss ways in which partnerships or projects have continued or endured despite challenges.	"My time has been taxed by all of this as well. I just don't have as much time and energy to devote to the project as I did before. Gratefully, they seem to be hanging in there with me and understanding." [Participant ID Redacted]
**Personal circumstances/ wellbeing**	Memoing	Apply this code when interviewee discusses anyone's (their own, stakeholder partners, research team, etc.) personal circumstances during the pandemic (related to personal life, work, etc.) or that they carry into the pandemic. Can be double coded with challenges, facilitators, etc. or impact of project/partnership on their wellbeing.	"Suddenly, I went from having full-time childcare to having all three of my children at home and having to facilitate online schooling for a three-year-old while still continuing to teach my classes and do my work." [Participant ID Redacted]
**Regulatory**	Pre-determined (guide)	Apply this code when interviewee discusses anything related to IRB or university policy.	"...for a while, we weren't allowed to go there. Even if I had wanted to, we couldn't have gone there. The IRB wouldn't have allowed it. That's one of the reasons." [Participant ID Redacted]
**Silver linings**	Memoing	Apply this code when participants discussed things that worked better, personally or in the work, bc of COVID-related circumstances; or something that turned out for the better/best even if unexpected.	"I knew that I couldn't push the community forward. If I'd pushed them, then I think it would've ruined the partnership. Also, it was a godsend to me because if they had been in a place where they could have continued to move forward, I could not have." [Participant ID Redacted]
**Virtual engagement**	Pre-determined (guide)	Apply this code when participants discuss experiences or perceptions of virtual engagement methods (e.g., Zoom, email), including issues related to virtual communication.	"Even though they all have smartphones, a lot of them have plans where data is very expensive. They don't wanna turn their camera on, and I don't wanna ask them to. Or they don't wanna use their minutes. We do a lot of texting, actually, with the mothers who joined our board." [Participant ID Redacted]

**Financial (Removed)**	Pre-determined (guide)	Apply this code when the interviewee discusses anything related to funder practices, compensation, existance of funds, fiscal issues, etc.	Note: Separated into compensation, funder/ing, and other during coding transcript 1
